# Cytoskeleton Remodeling-Related Proteins Represent a Specific Salivary Signature in PSC Patients

**DOI:** 10.3390/molecules29235783

**Published:** 2024-12-07

**Authors:** Elisa Ceccherini, Antonio Morlando, Francesco Norelli, Barbara Coco, Massimo Bellini, Maurizia Rossana Brunetto, Antonella Cecchettini, Silvia Rocchiccioli

**Affiliations:** 1 Institute of Clinical Physiology, National Research Council, 56124 Pisa, Italy; antoniomorlando@cnr.it (A.M.); francesco.norelli@cnr.it (F.N.); antonella.cecchettini@unipi.it (A.C.); 2 Hepatology Unit, Reference Centre of the Tuscany Region for Chronic Liver Disease and Cancer, University Hospital of Pisa, 56124 Pisa, Italy; b.coco@ao-pisa.toscana.it (B.C.); maurizia.brunetto@unipi.it (M.R.B.); 3 Gastrointestinal Unit, Department of Translational Research and New Technologies in Medicine and Surgery, University of Pisa, 56124 Pisa, Italy; massimo.bellini@unipi.it; 4 Department of Clinical and Experimental Medicine, University of Pisa, 56126 Pisa, Italy

**Keywords:** primary sclerosing cholangitis, primary biliary cholangitis, saliva, proteomics, LC-MS/MS, cytoskeleton

## Abstract

Primary sclerosing cholangitis (PSC) and Primary biliary cholangitis (PBC) are chronic inflammatory biliary diseases characterized by progressive damage of the bile ducts, resulting in hepatobiliary fibrosis and cirrhosis. Currently, specific biomarkers that allow to distinguish between PSC and PBC do not exist. In this study, we examined the salivary proteome by carrying out a comprehensive and non-invasive screening aimed at highlighting possible quali-quantitative protein deregulations that could be the starting point for the identification of effective biomarkers in future. Saliva samples collected from 6 PBC patients were analyzed using a liquid chromatography–tandem mass spectrometry technique, and the results were compared with those previously obtained in the PSC group. We identified 40 proteins as significantly deregulated in PSC patients compared to the PBC group. The Gene Ontology and pathway analyses highlighted that several proteins (e.g., small integral membrane protein 22, cofilin-1, macrophage-capping protein, plastin-2, and biliverdin reductase A) were linked to innate immune responses and actin cytoskeleton remodeling, which is a critical event in liver fibrosis and cancer progression. These findings provide new foundations for a deeper understanding of the pathophysiology of PSC and demonstrate that saliva is a suitable biological sample for obtaining proteomic fingerprints useful in the search for biomarkers capable of discriminating between the two cholestatic diseases.

## 1. Introduction

Primary sclerosing cholangitis (PSC) and primary biliary cholangitis (PBC) are chronic liver diseases characterized by inflammation and progressive damage of the bile ducts, resulting in hepatobiliary fibrosis and cirrhosis. PSC is a rare disease more frequent in men than in women (ratio 2:1) and shows a peak incidence around 40 years [1]. On the other hand, PBC predominantly affects women (ratio 1:9) with a peak range between 60 and 79 years [2]. These cholestatic diseases share multiple clinical (e.g., fatigue, pruritus, and jaundice) and biochemical features (elevated levels of alkaline phosphatase (ALP) and gamma-glutamyl transferase (GGT)), making differential diagnosis a challenge [3]. Histological examinations can provide additional information, showing the typical ‘onion skin’ lesions in PSC [4], while PBC displays lymphocytic infiltrates and granulomas surrounding the small bile ducts [5]. However, liver biopsies are very invasive and risky, therefore they are unattractive diagnostic tools in clinical routine. Thus, reliable and non-invasive biomarkers that can accurately distinguish between PSC and PBC, or can assess disease activity and prognosis, represent an urgency for patients and a challenge for researchers and clinicians.

Recent advances in proteomics mass spectrometry (MS)-based analyses are promising in this area, as they can provide a comprehensive profile of the protein content within biological samples such as serum, bile, or saliva, showing the alterations in protein expression that accompany a particular disease [6]. This approach has occasionally been applied in PSC and PBC patients to identify discriminatory proteins towards healthy individuals and patients with liver and/or biliary tumors or autoimmune liver disease. For instance, bile, serum, and urine MS-based proteomics were investigated to discriminate PSC from cholangiocarcinoma (CCA) and benign biliary disorders [7,8,9,10] or to identify proteins with potential prognostic value [11]. MS techniques were also applied to identify serum proteins able to discriminate PBC patients from healthy subjects [12] or autoimmune hepatitis patients [13]. Over the last 10 years, human saliva has been investigated for diagnostic purposes because it reflects the general health status of an individual [14,15]. Many studies have shown that saliva is a useful biofluid for detecting oral diseases [16,17] and also systemic conditions, including heart disease [18,19], diabetes [20], and several types of cancer [21,22,23,24]. In the hepatic field, some evidence suggested that liver function parameters can be assessed in saliva [25] and proposed several metabolites to discriminate between healthy individuals and patients with liver cirrhosis or hepatocellular carcinoma [26]. Interestingly, salivary proteomics investigations were carried out to identify salivary protein differences between subjects affected by PBC and autoimmune hepatitis [27,28], and between PSC patients and healthy individuals [29]. In this scenario, our study is the first to have conducted a comparative salivary proteomic analysis between subjects with PSC and PBC, providing new foundations for a deeper understanding of the PSC pathophysiology, and therefore it might have relevant implications for improving diagnosis, prognosis, and treatment strategies.

## 2. Results

### 2.1. Salivary Proteomics of PSC Patients

**Figure 1:**
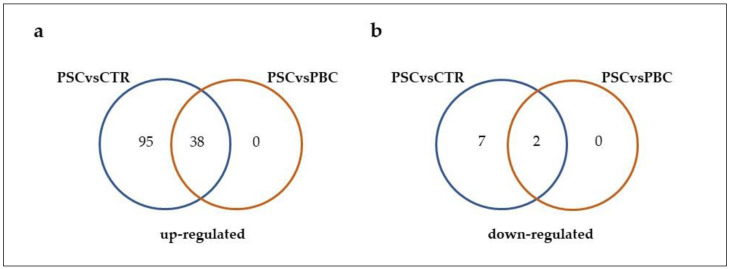
Venn diagrams showing the number of up-regulated (**a**) and down-regulated (**b**) proteins in PSC patients compared to both healthy subjects and PBC patients. Each colored circle represents a different dataset, and areas of overlap indicate shared proteins. The cutoff criteria for statistical significance were defined based on log_2_FC ≤ 0.58 or log_2_FC ≥ 0.58, and *p*-value < 0.05.

Saliva samples from PSC patients and healthy controls were compared, and, 142 proteins passed the requirement of having a Mann–Whitney *p*-value of less than 0.05 and log_2_FC ≤ 0.58 or log_2_FC ≥ 0.58 [29]. Among them, the expression levels of 40 proteins were also deregulated in PSC patients in respect to those in the PBC group (Figure 1 and Figure S1). As reported in Figure 1a, 38 proteins were up-regulated in PSC patients compared to both healthy individuals and PBC patients, and 2 proteins were down-regulated (Figure 1b) in PSC patients compared to both healthy and PBC subjects. In Table 1, we listed these proteins, and for each one, the log_2_FC and the *p*-value were reported. The protein with the highest FC was small integral membrane protein 22 (log_2_FC = 3.589). Multiple other proteins also displayed significant positive change in expression between the two groups, such as biliverdin reductase A (log_2_FC = 3.117), F-box only protein 50 (log_2_FC = 2.814), and glycogenin-1 (log_2_FC = 2.609). In addition, in the PSC group we detected a reduction in expression level for prosaposin and immunoglobulin lambda variable 3–10 when compared with those determined in PBC patients, with a log_2_FC of −0.779 and −0.615, respectively. In addition, 102 proteins showed significant change in their abundance in PSC subjects compared to healthy controls, but no change was identified with respect to the PBC group (Figure 1). In particular, we identified 7 down-regulated and 95 up-regulated proteins that are listed in Table S1. In Table 2, we listed the 7 down-regulated and the top 20 out of 95 up-regulated DEPs in PSC subjects compared to healthy controls, which showed no significant change compared to the PBC group, and for each one the log_2_FC and the *p*-value were reported. Among the down-regulated proteins, the serum amyloid P-component exhibited the lower FC (log_2_FC = −1.079), followed by Mucin-5B (log_2_FC = −0.980), Mucin-16 (log_2_FC = −0.936), Cystatin-A (log_2_FC = −0.812), Zinc-alpha-2-glycoprotein (log_2_FC = −0.781), WAP four-disulfide core domain protein 2 (log_2_FC = −0.700), and Immunoglobulin kappa constant (log_2_FC = −0.584). On the other hand, the protein that displayed the highest FC was Kallikrein-6 (log_2_FC = 5.136). Several other proteins also displayed up-regulation in the PSC group without significant change if compared to the PBC subjects, such as Protein S100-A7 (log_2_FC = 2.793), Aldo-keto reductase family 1 member B10 (log_2_FC = 2.513), Complement factor I (log_2_FC = 2.309), Low-affinity immunoglobulin gamma Fc region receptor III-A (log_2_FC = 2.231), and Osteoclast-stimulating factor 1 (log_2_FC = 2.218).

**Figure 2:**
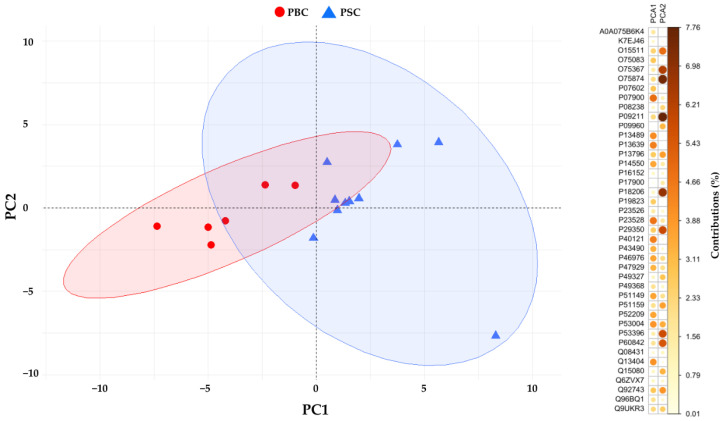
PCA scores plot with 95% confidence ellipse of the first two principal components (PC) obtained by the analysis of proteins significantly different in abundance (*p* < 0.05) between PSC patients (blue triangles) and PBC patients (red circles). For each protein, the contribution in the calculation of the variability of the first two principal components is expressed as a percentage.

All the 40 identified DEPs were subjected to Principal Component Analysis (PCA), and the reduced dataset was used to investigate the usability of these proteins as more specific for PSC status (Figure 2). The first two principal components (PC1 and PC2) explain 38.4% and 17.1% of the data variance, respectively. Among the two components, the first one contains the highest percentage of variance between the data, and itis of interest in discriminating between the two groups of patients.

### 2.2. Gene Ontology Enrichment and Pathway Analysis

**Figure 3:**
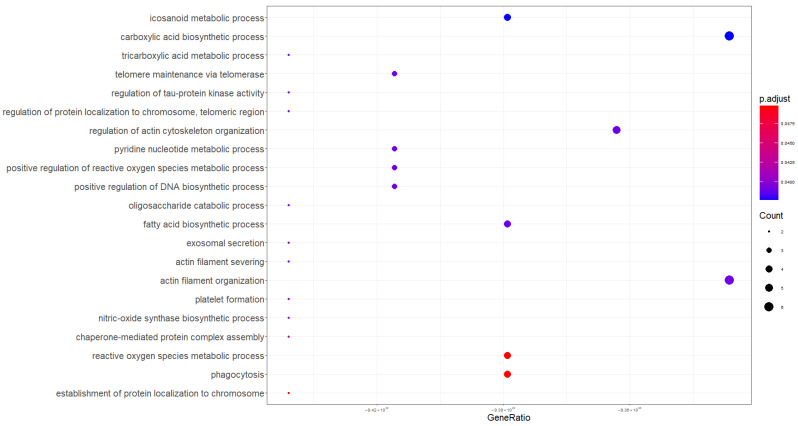
Dot plot showing enrichment of Gene Ontology Biological Processes (PBs) for the DEPs identified in PSC patients compared to both healthy control and PBC subjects.

**Figure 4:**
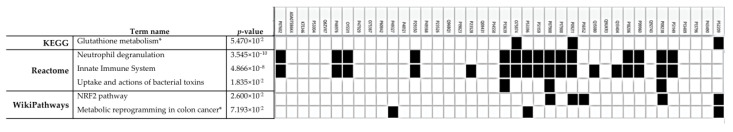
Pathway analysis via the online tool g:Profiler. For each term, the statistical significance and the involved proteins are reported. * Terms not statistically significant but biologically relevant.

To obtain more biological insights, the GO enrichment analysis was performed using the clusterProfiler V.4.8.3 R package. Firstly, GO analysis was performed on the proteins listed in Table 1 to identify the changes in the BPs more tightly modulated by the PSC. As shown in Table 3 and Figure 3, the BPs were primarily enriched in biosynthetic and metabolic processes involving carboxylic acid, eicosanoids, fatty acids, and pyridine nucleotides. In addition, processes related to the organization of actin filaments, platelet formation, and the reactive oxygen species metabolic process also emerged. For deeper investigation, a pathway analysis was performed using g:Profiler, discovering significant enriched terms related to the innate immune responses and neutrophils degranulation (Figure 4). This analysis also highlighted two terms not statistically significant but biologically relevant, identified as metabolic reprogramming in colon cancer and glutathione metabolism. PSC and PBC are both chronic liver diseases involving bile ducts, sharing several overlapping clinical and serological features, though they are distinct pathologies. In order to highlight possible biological processes common to both diseases, a GO analysis was conducted on Table S1 proteins, which are deregulated in the PSC compared to the control group but not significantly different in expression compared to subjects with PBC. As shown in Table 4, GO enrichment analysis revealed that the protein-related BPs were markedly concentrated in inflammatory responses, phagocytosis, and cellular redox homeostasis (GO:0072593, GO:1990748, GO:0042744).

## 3. Discussion

In this explorative study, we have analyzed and compared the saliva proteomic profiles of PBC patients with those obtained in our previous study on PSC subjects and healthy controls [29]. Comparative analysis showed 142 DEPs in the PSC group compared to healthy subjects, and among them, 102 proteins did not have significantly different levels with respect to the PBC group (Table 2), suggesting that these proteins are not good discriminators between the two cholestatic diseases. Interestingly, 40 of the 142 proteins might be more indicative of PSC, having shown significant deregulation compared to patients with PBC (Table 1). For these proteins we performed a GO analysis highlighting 7 proteins as significantly related to the actin filaments organization (Table 3; Figure 3). Among them, the most up-regulated protein was small integral membrane protein 22, with a log_2_FC = 3.589. Although no literature data reported information on its involvement in cholestatic diseases or related manifestations, Polycarpou-Schwarz and colleagues demonstrated that small integral membrane protein 22 is involved in actin cytoskeleton organization in the MCF7 breast cancer cell line, affecting cell proliferation, cell migration, and cell cycle progression [30]. Cofilin-1 is an actin-binding protein that regulates the cytoskeleton dynamics in hepatic stellate cells (HSCs), triggering the deposition of type I collagen during liver fibrosis [31]. Although the up-regulation of cofilin-1 was only detected in another salivary proteomics study on patients with PBC [27], two recent studies demonstrated the increase in cofilin-1 expression in hepatocellular carcinoma tissues [32,33]. Macrophage-capping protein is a calcium-sensitive member of the gelsolin family and is involved in the remodeling of the cytoskeleton actin filaments [34]. No clinical study has shown its deregulation in biological fluids or tissues from patients with PSC or PBC; however, the association of increased macrophage-capping protein expression with vascular invasion in patients with cholangiocarcinoma [35] and hepatocellular carcinoma tissues [36] was recently demonstrated. Another deregulated salivary protein related to cytoskeleton shaping was actin-related protein 2/3 complex subunit 5 that modulates the actin polymerization after the stimulation by nucleation-promoting factor [37]. Huang and colleagues highlighted higher actin-related protein 2/3 complex subunit 5 expression in hepatocellular carcinoma tissues and cells when compared with healthy liver tissues or normal liver cells [38]. Although our results are preliminary and supporting literature data are scarce, they seem to suggest the proteins discussed above as possible salivary indicators of liver fibrosis caused by actin cytoskeleton remodeling in HSCs. Furthermore, their up-regulation in hepatocellular carcinoma and cholangiocarcinoma would suggest that these proteins could be indicative of the progression of cholestatic disease towards a cancerous form. Among the proteins linked to cytoskeletal shaping, plastin-2 is the one with the lowest degree of deregulation in PSC patients compared to the PBC group (log_2_FC = 0.716). Tit-Oon and colleagues analyzed the secretoma of cholangiocarcinoma cells cultured in 3D, identifying plastin-2 as a secreted protein; protein not found in mixed hepatocarcinoma-cholangiocarcinoma cell culture [39]. Joshi and colleagues have recently demonstrated the connection between plastin-2 and the NLRP3 inflammasome activation in macrophages using a mouse model of lung fibrosis [40]. It is well known that increased NLRP3 inflammasome expression was a key event in HSC activation and in cholangiocytes switch to a reactive phenotype, contributing to the development of liver and biliary fibrosis [41,42,43]. Consistent results were observed in liver sections of patients affected by PSC [41] and in a Mdr2-knockout mouse model [44]. These findings are very interesting as they would suggest a more pronounced relationship of plastin-2 with the biliary tissue rather than the liver parenchyma, corroborating its salivary up-regulation as related to biliary fibrosis in PSC, in which macrophages play a major role [45]. Considering the secretion of plastin-2 by cholangiocarcinoma cells, our results could suggest this protein as a salivary indicator of a probable evolution towards cancer. Although biliverdin reductase A did not emerge as being significantly associated with any biological process in our GO analysis, we believe it may have considerable clinical relevance. Very little is known about the role of biliverdin reductase A in the pathogenesis and progression of cholestatic diseases; however, according to our results, recent studies highlighted its up-regulation in serum samples from PSC patients [46]. Biliverdin reductase A is an enzyme that converts biliverdin into bilirubin, which possesses cytoprotective properties in response to oxidative stress and regulates the innate immune responses [47,48,49]. Recently, Weaver and colleagues suggested that biliverdin reductase A may prevent HSCs fibrogenesis by antagonizing the toll-like receptor 4 (TLR4) signaling pathway [50]. Indeed, TLR4 signaling plays a key role in activated HSCs, which represent the major fibrogenic cell type during liver fibrosis [43,51]. In addition, increased biliverdin reductase A expression was detected on the plasma membrane of macrophages after lipopolysaccharide treatment, used to initiate an inflammatory response [52]. Collectively, this evidence would attribute to biliverdin reductase A a key role in the activation of HSCs by actively contributing to the restraint of the fibrotic process. In this perspective, the increased salivary expression of biliverdin reductase A in patients with PSC could be indicative of its cytoprotective activity against liver fibrosis. In this study, we demonstrated that PSC significantly alters the salivary proteome compared to PBC and healthy controls, identifying multiple cytoskeleton-related proteins that might represent a specific signature of the PSC. To corroborate our results, all the 40 DEPs were subjected to PCA, and the reduced dataset was used to investigate if these proteins were more specific for PSC status (Figure 2). Our analysis had shown that the expression of four proteins (biliverdin reductase A, macrophage-capping protein, heat shock protein HSP 90-beta, and cofilin-1) out of the 40 salivary DEPs might be more indicative of PSC than the PBC group. These data would seem to support the results of the pathway analysis (Figure 4) that showed a greater involvement of the innate immune system in PSC than in PBC subjects. Indeed, cofilin, macrophage-capping protein, and HSP90 are proteins whose role in modulating the innate immune response by macrophages is well documented [53,54,55,56]. In addition, previous studies showed a higher number of macrophages in liver biopsies from patients with PSC than in those from PBC or other liver diseases [57,58]. This phenomenon could underlie the greater involvement of innate immune system responses in patients with PSC than in the other groups analyzed in this study, suggesting that the salivary proteins macrophage-capping protein, cofilin-1, and heat shock protein HSP 90-beta could be indicators of the macrophage involvement in the pathophysiology of PSC. Since some of the proteins discussed (e.g., actin-related protein 2/3 complex subunit 5, cofilin-1,plastin-2 and macrophage-capping protein) go into post-translational modifications (PMTs), such as phosphorylation and glycosylation, that regulate their activity, distribution, and stability [59,60,61,62,63,64], further MS-based PTM analyses are needed to clarify the role that might have in the onset and progression of PSC [65,66]. Certainly, the most impactful limitation of this study is related to the small patient cohort that should be addressed in larger population studies to confirm the peculiar phenomena observed. However, this preliminary exploratory study provides important information for a deeper understanding of the pathophysiology of PSC, demonstrating that saliva is a suitable biological sample for obtaining proteomic fingerprints useful in the search for biomarkers that can effectively discriminate between the two cholestatic diseases.

## 4. Materials and Methods

### 4.1. Reagents

All the reagents and materials used for sample collection, processing and analysis in liquid chromatography–tandem mass spectrometry (LC-MS/MS) were detailed in our previous article [67].

### 4.2. Patient Characteristics

Six female patients with confirmed PBC were subsequently recruited at the University Hospital of Pisa (Hepatology Unit and Gastroenterology Unit), according to the established inclusion criteria. The patient group was in a range of 52–70 years (median age of 60.5 years). A saliva sample was obtained from each subject as previously described [29,68]. At the time of saliva collection, patients showed a median value for alanine aminotransferase (ALT), aspartate aminotransferase (AST), and total bilirubin of 17.00 U/L (10–48), 18.00 U/L (18–42), and 0.36 mg/dL (0.22–0.49), respectively. The median GGT, ALP, albumin, and gamma globulin values were 79 U/L (12–161), 123 U/L (84–350), 4.35 g/L (3.8–5.0), and 19.7% (13.9–24.6), respectively. A total of 2 patients out of 6 were positive for anti-nuclear antibodies (ANA) and anti-mitochondrial antibodies (AMA). Among them, three patients were positive for ANA and P-ANCA, and just one patient was positive for anti-smooth muscle antibodies (ASMA). A total of 6 males and 4 females were enrolled for both the PSC group and healthy individuals, aged between 18 and 70 years (median age of 49 years) and 27–65 years (median age of 41 years), respectively.

The study obtained the approval of the Comitato Etico Area Vasta Nord Ovest (Protocol code 57532, 30 October 2019, Pisa, Italy). All the patients signed an informed consent to participate in the study.

### 4.3. Saliva Collection, Processing, and LC-MS/MS Analysis

Spontaneous saliva from each PBC patient was collected and enriched in extracellular vesicles by applying a differential centrifugation isolation protocol; then the proteins were extracted and quantified according to our previous papers [29,68]. A total of 100 µg of proteins for each sample were subsequently reduced, alkylated, and digested. After that, the peptide mixtures were centrifuged, desalted, resuspended in CH_3_CN/0.1% HCOOH (ratio 5/95) to achieve a final peptide concentration of 2 µg/µL, and analyzed by LC-MS/MS as previously reported [29]. Sample analysis was performed according to our previous paper [29] using a micro-HPLC (Eksigent Ekspert microLC 200, AB Sciex, Concord, ON, Canada) coupled with a Triple TOF 5600 mass spectrometer (AB Sciex, Concord, ON, Canada). The protein quantification was achieved through Sequential Window Acquisition of all Theoretical fragment ion Mass Spectra (SWATH-MS) methodology, as previously reported [29].

### 4.4. Data Processing and Statistical Analysis

SWATH raw files were processed using the free universal software DIA-NN (version 1.8) as previously described [29], quantifying 733 proteins. This dataset was compared with that obtained from our previous salivary proteomic analysis on patients with PSC and healthy controls [29]. For each protein, we compared the fold change (FC) calculated as the ratio between the mean of abundance in PSC patients and the PBC group with those obtained as the ratio between the mean of abundance in PSC patients and the healthy control group. Proteins were considered significantly differentially expressed (DEPs) when FC ≤ 1/1.5 or FC ≥ 1.5, and *p*-value < 0.05 (determined using the non-parametric Wilcoxon test).

The DEPs were also subjected to Gene Ontology (GO) over-representation analysis, which was performed using the clusterProfiler package in R (version 4.8.3) and the OrgDb annotation database, Genome-wide annotation for Human (version 3.17.0). Significant GO terms were identified using a *p*-value threshold of 0.05, with False Discovery Rate (FDR) correction applied via the Benjamini–Hochberg method. The pathway analysis was performed using the g:Profiler tool [69]. Principal component analysis (PCA) was performed using the FactoMineR package implemented in R (version 2.11). To highlight the variables that most contributed to each dimension, the corrplot package (version 0.92) was used.

## Figures and Tables

**Table 1:** List of 40 DEPs in PSC patients compared to both healthy and PBC subjects, which passed the cutoff log_2_FC ≤ 0.58 or log_2_FC ≥ 0.58 FC, and a Mann–Whitney *p*-value < 0.05.

Protein ID	Protein Name	log_2_FC(PSCvsPBC)	*p*-Value
**DOWN-REGULATED PROTEINS**			
P07602	Prosaposin	−0.779	0.006
A0A075B6K4	Immunoglobulin lambda variable 3–10	−0.615	0.004
**UP-REGULATED PROTEINS**			
K7EJ46	Small integral membrane protein 22	3.589	0.002
P53004	Biliverdin reductase A	3.117	0.0001
Q6ZVX7	F-box only protein 50	2.814	0.006
P46976	Glycogenin-1	2.609	0.001
O15511	Actin-related protein 2/3 complex subunit 5	2.370	0.017
P47929	Galectin-7	2.267	0.0001
O75367	Core histone macro-H2A.1	2.225	0.015
P60842	Eukaryotic initiation factor 4A-I	2.096	0.013
P49327	Fatty acid synthase	2.058	0.011
P40121	Macrophage-capping protein	1.995	0.001
P29350	Tyrosine-protein phosphatase non-receptor type 6	1.968	0.008
P49368	T-complex protein 1 subunit gamma	1.714	0.002
P23526	Adenosylhomocysteinase	1.543	0.005
Q96BQ1	Protein FAM3D	1.503	0.005
P19823	Inter-alpha-trypsin inhibitor heavy chain H2	1.464	0.015
P23528	Cofilin-1	1.413	0.001
Q08431	Lactadherin	1.358	0.016
P14550	Aldo-keto reductase family 1 member A1	1.309	0.021
P13639	Elongation factor 2	1.195	0.0002
O75874	Isocitrate dehydrogenase [NADP] cytoplasmic	1.190	0.042
P53396	ATP-citrate synthase	1.183	0.041
P51159	Ras-related protein Rab-27A	1.159	0.014
P07900	Heat shock protein HSP 90-alpha	1.140	0.013
P17900	Ganglioside GM2 activator	1.120	0.042
P09211	Glutathione S-transferase P	1.102	0.009
P16152	Carbonyl reductase [NADPH] 1	1.051	0.039
Q15080	Neutrophil cytosol factor 4	1.012	0.045
Q9UKR3	Kallikrein-13	0.996	0.032
Q13404	Ubiquitin-conjugating enzyme E2 variant 1	0.960	0.0120
P18206	Vinculin	0.953	0.038
P09960	Leukotriene A-4 hydrolase	0.892	0.043
Q92743	Serine protease HTRA1	0.840	0.033
P08238	Heat shock protein HSP 90-beta	0.825	0.020
P51149	Ras-related protein Rab-7a	0.807	0.003
P13489	Ribonuclease inhibitor	0.798	0.021
P13796	Plastin-2	0.716	0.045
P43490	Nicotinamide phosphoribosyltransferase	0.654	0.021
P52209	6-phosphogluconate dehydrogenase, decarboxylating	0.625	0.004

**Table 2:** List of the 7 down-regulated and the top 20 out of 95 up-regulated DEPs identified in PSC subjects compared to healthy controls, which showed no significant change compared to the PBC group. For each protein, the cutoff of log_2_FC ≤ 0.58 or log_2_FC ≥ 0.58, and a Mann–Whitney *p*-value < 0.05 were applied.

Protein ID	Protein Name	log_2_FC(PSCvsControl)	*p*-Value
**DOWN-REGULATED PROTEINS**			
P02743	Serum amyloid P-component	−1.079	0.013
Q9HC84	Mucin-5B	−0.980	0.0002
Q8WXI7	Mucin-16	−0.936	0.031
P01040	Cystatin-A	−0.812	0.034
P25311	Zinc-alpha-2-glycoprotein	−0.781	0.021
Q14508	WAP four-disulfide core domain protein 2	−0.700	0.037
P01834	Immunoglobulin kappa constant	−0.584	0.048
**UP-REGULATED PROTEINS**			
Q92876	Kallikrein-6	5.136	0.006
P31151	Protein S100-A7	2.793	0.003
O60218	Aldo-keto reductase family 1 member B10	2.513	0.048
P05156	Complement factor I	2.309	0.017
P08637	Low affinity immunoglobulin gamma Fc region receptor III-A	2.231	0.012
Q92882	Osteoclast-stimulating factor 1	2.218	0.009
P19878	Neutrophil cytosol factor 2	2.096	0.005
O75923	Dysferlin	2.018	0.006
Q96QK1	Vacuolar protein sorting-associated protein 35	1.916	0.01
Q14002	Carcinoembryonic antigen-related cell adhesion molecule 7	1.897	0.045
Q13393	Phospholipase D1	1.865	0.001
P53618	Coatomer subunit beta	1.822	0.02
Q9BQR3	Serine protease 27	1.771	0.015
Q92839	Hyaluronan synthase 1	1.757	0.023
P30101	Protein disulfide-isomerase A3	1.683	0.001
Q6JEL2	Kelch-like protein 10	1.678	0.018
O43240	Kallikrein-10	1.603	0.002
Q8N6Q3	CD177 antigen	1.548	0.006
Q9Y2X7	ARF GTPase-activating protein GIT1	1.507	0.001
Q14974	Importin subunit beta-1	1.503	0.015

**Table 3:** GO enrichment analysis of the DEPs identified in PSC patients compared to both healthy control and PBC subjects. The GO terms belonging to BPs are listed, and for each one the significance level (*p*-adjust < 0.05) and the gene list were reported.

ID	Description	*p*-Adjust	Gene ID
GO:0046394	carboxylic acid biosynthetic process	0.0387	GSTP1/LTA4H/AKR1A1/CBR1/FASN/ACLY
GO:0006690	eicosanoid metabolic process	0.0377	CES2/GSTP1/LTA4H/CBR1
GO:0007015	actin filament organization	0.0390	ARPC5/WDR1/RNH1/LCP1/CFL1/CAPG
GO:0032956	regulation of actin cytoskeleton organization	0.0390	SMIM22/ARPC5/WDR1/RNH1/CAPG
GO:0006633	fatty acid biosynthetic process	0.0390	GSTP1/CBR1/FASN/ACLY
GO:0007004	telomere maintenance via telomerase	0.0390	HSP90AA1/HSP90AB1/CCT3
GO:2000379	positive regulation of reactive oxygen species metabolic process	0.0390	GSTP1/CBR1/RAB27A
GO:2000573	positive regulation of DNA biosynthetic process	0.0390	HSP90AA1/HSP90AB1/CCT3
GO:0019362	pyridine nucleotide metabolic process	0.0390	IDH1/NAMPT/PGD
GO:0072350	tricarboxylic acid metabolic process	0.0390	IDH1/ACLY
GO:1902947	regulation of tau-protein kinase activity	0.0390	HSP90AA1/HSP90AB1
GO:1904814	regulation of protein localization to chromosome, telomeric region	0.0390	MACROH2A1/CCT3
GO:0051014	actin filament severing	0.0390	CFL1/CAPG
GO:0009313	oligosaccharide catabolic process	0.0390	GM2A/PGD
GO:1990182	exosomal secretion	0.0390	RAB7A/RAB27A
GO:0030220	platelet formation	0.0406	WDR1/PTPN6
GO:0051767	nitric-oxide synthase biosynthetic process	0.0406	GSTP1/NAMPT
GO:0051131	chaperone-mediated protein complex assembly	0.0414	HSP90AA1/HSP90AB1
GO:0006909	phagocytosis	0.0498	RAB7A/RAB27A/MFGE8/NCF4
GO:0072593	reactive oxygen species metabolic process	0.0498	GSTP1/CBR1/RAB27A/NCF4
GO:0070199	establishment of protein localization to chromosome	0.0498	MACROH2A1/CCT3

**Table 4:** GO enrichment analysis of the DEPs identified in PSC patients compared to healthy control, which showed no significant change compared to the PBC group. The GO terms belonging to BPs are listed, and for each one the significance level (*p*-adjust < 0.05) and the gene list were reported.

ID	Description	*p*-Adjust	Gene ID
GO:0006909	phagocytosis	0.0028	DYSF/AHSG/ANXA3/CYBA/NCF2/ICAM3/ANXA11/ PTPRJ/PYCARD
GO:0072593	reactive oxygen species metabolic process	0.0028	HBD/HBB/G6PD/CYBA/ARF4/NCF2/PRDX5/CD177/ DHRS4
GO:0007596	blood coagulation	0.0308	KNG1/HBB/SERPING1/SAA1/GNA13/FERMT3/TLN1
GO:0006098	pentose-phosphate shunt	0.0308	H6PD/G6PD/PGAM1
GO:0006740	NADPH regeneration	0.0308	H6PD/G6PD/PGAM1
GO:0052547	regulation of peptidase activity	0.0329	KNG1/AHSG/SERPING1/MMP9/CD44/PRDX5/PSME1/ ITIH4/PYCARD
GO:0002526	acute inflammatory response	0.0329	IGHG1/AHSG/FCGR3A/SAA1/ITIH4
GO:1990748	cellular detoxification	0.0329	AKR1B10/HBD/HBB/PRDX6/PRDX5
GO:0042060	wound healing	0.0347	KNG1/HBB/SERPING1/ANXA6/SAA1/CD44/GNA13/ FERMT3/TLN1
GO:0051156	glucose 6-phosphate metabolic process	0.0347	H6PD/G6PD/PGAM1
GO:0042744	hydrogen peroxide catabolic process	0.0383	HBD/HBB/PRDX5
GO:0050878	regulation of body fluid levels	0.0385	KNG1/HBB/SERPING1/SAA1/CYBA/GNA13/FERMT3/ TLN1
GO:0006958	complement activation, classical pathway	0.0428	SERPING1/CFI/C7
GO:0002437	inflammatory response to antigenic stimulus	0.0441	IGHG1/FCGR3A/IL1RN/IL36A
GO:0030212	hyaluronan metabolic process	0.0471	CD44/ITIH4/HAS1

## Data Availability

Data will be made available on request.

## Supplementary Materials

The following supporting information can be downloaded at: https://www.mdpi.com/article/10.3390/molecules29235783/s1, Table S1: List of down and up-regulated proteins identified in PSC patients compared to healthy control which showed no significant change compared to the PBC group. The cutoff log_2_FC ≤ 0.58 or log_2_FC ≥ 0.58 FC, and a Mann–Whitney *p*-value < 0.05 were applied; Figure S1: Volcano plot of the significantly DEPs in PSC patients compared to both healthy and PBC subjects. The red dots represent the 38 up-regulated proteins, blue dots mean the 2 down-regulated proteins, and gray ones are non-significantly altered proteins. The x-axis represents the protein difference reported as log_2_FC, and the y-axis corresponds to the −log_10_(*p*-value).
