# Supplementary figures and images for: Cytoskeleton Remodeling-Related Proteins Represent a Specific Salivary Signature in PSC Patients
Source: Molecules. 2024 Dec 7;29(23):5783. doi: 10.3390/molecules29235783 (PMC11643721; doi:10.3390/molecules29235783)

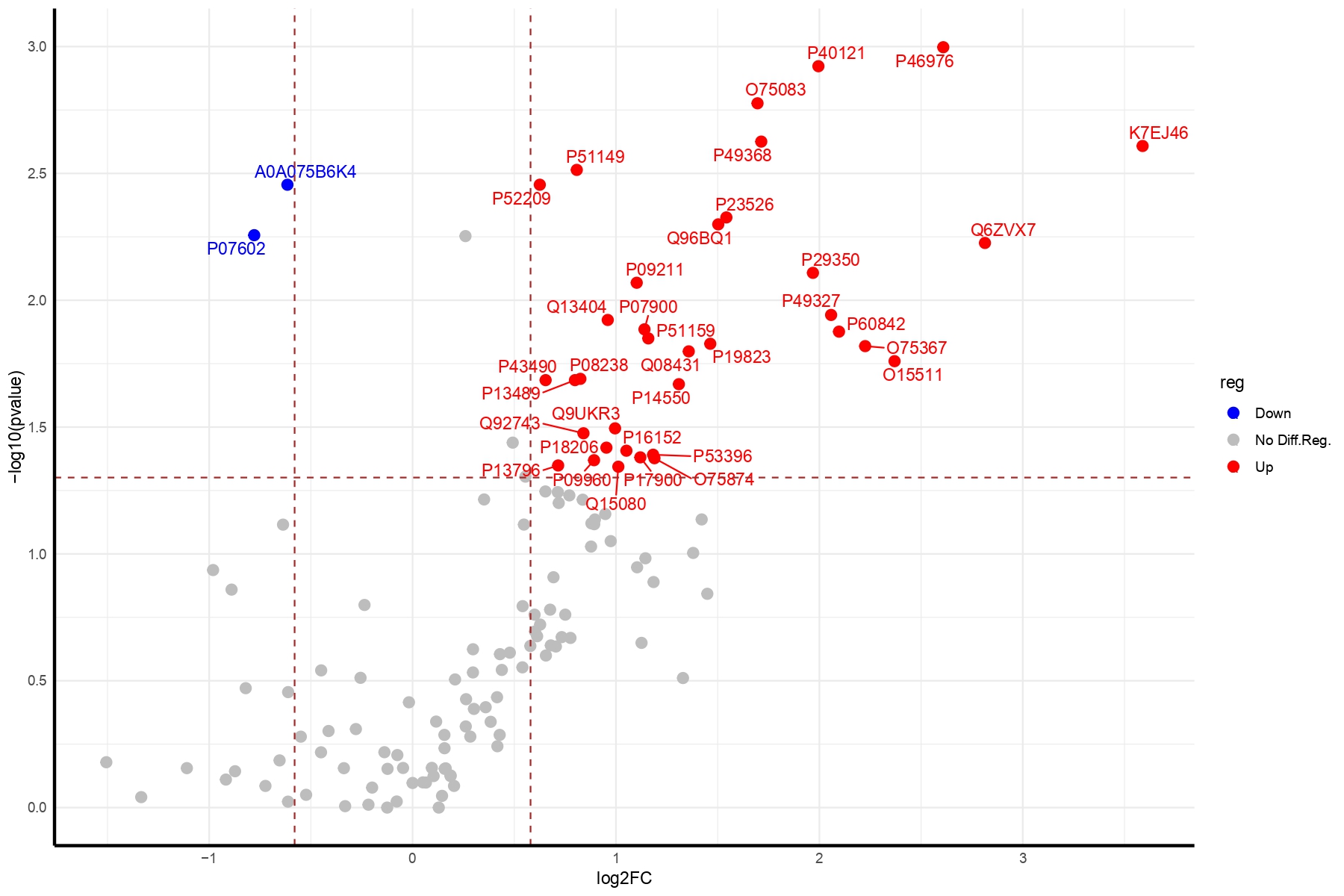

Supplement: Supplementary file 1 [file molecules-29-05783-s001.zip › Volcano Plot_Figure S1.jpg]
